# Exploring gender factors affecting women’s involvement in public health capacity development programmes in Nigeria

**DOI:** 10.1186/s12889-025-25438-6

**Published:** 2025-11-21

**Authors:** Ganiyat Eshikhena, Nwadiuto Ojielo, Adaeze Eche-George, Uche Ibe, Timiebiere Sabenus, Christian Abba, Alice Ogenyi, Sharon Uzoma, Charles Obi, Dupsy Akoma, Obonganwan Akpabio, Abayomi Amire, Nicholas Oyeh, Ifeoma Idigbe, Chijioke Kaduru

**Affiliations:** 1 Corona Management Systems, Abuja, Nigeria; 2 Nigeria Centre for Disease Control and Prevention (NCDC), Abuja, Nigeria; 3 Nigerian Institute of Medical Research (NIMR), Lagos, Nigeria

**Keywords:** Workforce, Capacity development, Gender, Equality, Barriers

## Abstract

**Introduction:**

Gender-sensitive workforce capacity development (WCD) is needed in the public health care system to advance global health, achieve universal health coverage, and accomplish sustainable development goals. Globally, there has been a progressive trend towards increased gender diversity within the public health workforce, however; gaps still exist especially in access to workforce capacity development opportunities for women. This study aimed to assess gender-related factors affecting women’s participation in workforce capacity development in public health space in Nigeria.

**Method:**

This study adopted a desk review and a qualitative approach to gain insights into the WCD challenges faced by women working in public health in Nigeria. The study duration was conducted between April 2023 and October 2023. Data were collected through a desk review of recent literature and from focused group discussions (FGDs), in-depth interviews (IDIs) and key informant interviews (KIIs). Recordings from all the interviews and FGDs were transcribed verbatim, coded, and analysed thematically.

**Results:**

Barriers to women’s participation in Health WCD included gender stereotyping, sexual harassment, marriage, family obligations, pregnancy and childbearing, lack of self-confidence, insecurity, socio-cultural and ethnic diversity, inconvenient timing and impromptu notice of opportunities, lack of funding and poor level of education. There are enablers- Gender-related policies and practices within the workspace, and strategies to limit the barriers which include awareness campaigns, financial incentives, a supportive environment, and workplace mentorship.

**Conclusion:**

The findings of this study revealed that women’s participation in health WCD is limited by gender stereotyping, socio-cultural and political barriers. This is in addition to poor implementation and practice of existing gender-related policies. Addressing these barriers would require increasing sensitisation and awareness campaigns to promote knowledge and implementation of available gender policies in workplaces, addressing socio-cultural norms, and providing a supportive workplace environment for women.

**Supplementary Information:**

The online version contains supplementary material available at 10.1186/s12889-025-25438-6.

## Introduction

A strong public health workforce is critical to achieving Universal Health Coverage (UHC) and health-related Sustainable Development Goals (SDGs) [1]. Yet, low- and middle-income countries (LMICs) face severe shortages, with an estimated 18 million additional health workers required by 2030 [2]. Meeting this need depends on workforce capacity development (WCD), the process of equipping individuals and institutions with the knowledge, skills, and resources necessary for effective and sustainable performance [1–3].In many LMICs, however, health workers depend on limited training and experiential learning, leaving gaps that compromise system resilience and population health outcomes [4, 5].The World Health Organization’s *Workforce 2030 Strategy* highlights that weak institutional support and inadequate training contribute to low preparedness across health systems [6]. Rowe et al. (2018) while conducting a systematic review on the effective strategies to improve health-care provider practices in low-income and middle-income countries, highlighted that health worker performance in LMICs remains suboptimal due to insufficient supervision, lack of continuing professional development, and limited access to updated clinical guidelines [7].

Addressing these systemic gaps requires not only stronger training mechanisms but also deliberate integration of gender considerations into WCD [8]. The World Bank continues to emphasise gender equality at all levels for equal distribution of opportunities and resources [8]. The importance of gender equality and the advantages of having women in the public health workforce cannot be overstated, with evidence showing better outcomes when women are fully engaged as healthcare providers [6]. Despite this, women represent 71% of the global health workforce yet hold only 30–35% of leadership positions [12]. This imbalance undermines diversity in decision-making, slows innovation, and restricts the system’s ability to respond to complex health challenges. Projections suggest that, at the current pace, gender parity in managerial roles will not be reached for more than 170 years [9, 10]. Closing this gap is not only a matter of fairness; it is also a public health imperative.

In Nigeria, these disparities are especially pronounced. A 2025 BMJ Global Health review found that women occupied only one-third of leadership roles in health institutions, with stark underrepresentation in senior decision-making positions [5, 11]. The USAID Health Workforce Management Gender Equality and Social Inclusion Analysis reported that the absence of workplace protections, such as sexual harassment policies and reporting systems, disproportionately constrains women’s participation in workforce development programs [12]. Furthermore, national labour statistics consistently show higher underemployment rates among women compared to men, reinforcing the structural barriers that limit career progression and leadership opportunities [13]. These inequities not only prevent women from fulfilling their professional potential but also weaken Nigeria’s capacity to address pressing health challenges.

Against this backdrop of persistent inequities, it becomes essential to generate context-specific evidence that can illuminate how gender dynamics shape workforce development in Nigeria. This study addresses a critical gap in qualitative research on gender and WCD in Nigeria. Specifically, it explores the gender-related factors that influence women’s participation in workforce development opportunities, the policies and practices that shape these experiences, and the strategies needed to overcome barriers. By doing so, the study aims to inform gender-sensitive approaches that can strengthen workforce development, enhance women’s leadership, and ultimately improve health outcomes.

## Method

### Study design

This study adopted desk reviews and a qualitative approach to identify gender-related factors influencing participation in WCD for women working in public health in different states (Bayelsa, Imo, Kano States and Abuja (FCT)) in Nigeria. These states were purposively selected to ensure representation across Nigeria’s major geopolitical zones: Bayelsa (South–South), Imo (South–East), Kano (North–West), and Abuja FCT (North–Central). This selection captures diversity in sociocultural contexts, health system structures, and gender norms, thereby strengthening the representativeness of the findings while acknowledging that not all six geopolitical zones could be covered.

Data collection and analysis were guided by the Social–Ecological Model (SEM) theoretical framework, which conceptualises influences on participation across multiple, interacting levels (individual, interpersonal/household, organisational, community, and policy/system) [14]. We used SEM to shape the interview guide and to organise analysis of barriers, enablers, strategies, and recommendations, recognising that women’s uptake of workforce capacity development (WCD) opportunities emerges from the interplay of these levels [14, 15]).

### Study setting

The study participants were recruited from the public sector (ministries, departments, and agencies of government) and private sector.

### Data collection and sampling strategy

#### Desk review

The desk review utilised a comprehensive search strategy to identify 59 relevant literatures, reports, and policy documents from academic databases (Google Scholar, WHO library databases, PubMed databases). The literature search utilised keywords on gender sensitivity issues in workforce capacity development in Nigeria like women’s participation in the health workforce, gender and public health workspace. The search query was tailored to the specific requirements of each database and the Boolean operators used are ‘OR’ and ‘AND’. Other searches from reports and policy documents on gender workforce capacity developments were retrieved. The inclusion criteria involved publications published between 2018 and 2023 written in English while duplicates of articles were excluded.

A total of 87 articles were identified from the literature search, 17 articles were excluded based on titles and duplicates, furthermore, 11 articles were excluded based on the objectives of the study. Finally, 59 articles including reports and policy documents were reviewed. These were published articles, reports, or policy documents.

#### Qualitative component

The qualitative component aimed to gather first-hand information on the barriers affecting women and offer solutions (recommendations). A total of 44 qualitative interview sessions were conducted, involving 72 participants. These included 23 key informant interviews (KIIs), four focus group discussions (FGDs) with eight participants per group (32 participants in total), and 17 in-depth interviews (IDIs) with women working in the public health sector. Participants were purposively selected from a diverse range of stakeholders, including government ministries, departments and agencies, private organisations, non-governmental organisations, and international institutions, to capture perspectives relevant to workforce capacity development and gender-related challenges in the public health space.

#### Data collection and analysis

The semi-structured interview guide included prompts at each SEM level (e.g., individual skills and confidence; household dynamics and caregiving; organisational policies and practices; community norms and safety; policy and programme contexts) to ensure comprehensive coverage [14, 15] Also, the interview guide was developed specifically for this study to explore gender-related factors affecting women’s involvement in public health capacity development programmes (see *Additional file 1* for the English version of the guide). This guide was used to collect data from participants who gave their consent. The interviews were conducted by a team of experienced data collectors. The interviews were conducted in English and Hausa in comfortable private rooms and their offices. The interview guide elicited responses on the barriers affecting women’s participation, enablers and recommendations. Audio recorders were used to collect the data from all the participants. Participants were called ahead of time to choose a suitable time for the interview and were compensated for their time with lunch money. Field notes that summarised salient points or highlighted thematic areas during the interviews were written as memos.

All the recordings collected from the interviews were transcribed verbatim. Manual transcription was done, and data was analysed using thematic analysis.

The interview transcripts were read repeatedly and coded to identify themes and sub-themes. Data was analysed utilising Thematic Analysis. The main themes were inputted as parent nodes while the sub-themes were entered as child nodes. Verbatim quotes were included to support the themes and sub-themes. We conducted reflexive thematic analysis and then mapped codes and themes to SEM levels, allowing us to examine how factors clustered and interacted across individual, household, organisational, community, and policy/system domains.

### Ethical approval

Ethics approval to conduct this study was obtained from* the *Health Research Ethics Committee (HREC) with approval numberNHREC/01/01/2007.Eligible participants were given a detailed written consent form containing information about the study. The consent forms explained the study process and their right to either decline or participate. Participants, who agreed to participate and gave their consent, signed a consent form and were interviewed.

## Results

Thematic findings were presented using the SEM levels as an organising scaffold, showing how barriers, enablers, and practical solutions operate and reinforce one another from the individual through to the system and policy level. A total of 72 respondents were interviewed. The participant’s demographics consisted of early-, mid-, and senior-career women in public health practice and from decision-makers and managers from public health agencies and institutions across the country, with the age group between 24–59 years in the different geopolitical zones in Nigeria. The majority were females (82%); the average age was 41 years, and they were predominantly Christians and Muslims. A greater proportion of them (75%) had a bachelor’s degree as a minimum educational qualification.

Key informant Interviews consisted of Decision makers and programme managers. In-depth Interviews consisted of early, mid-level, and senior career women. Focus Group Discussion 4 (FGDs) (12 participants each consisted of Community Health Extension Workers (CHEWs), < level 8 Nurses, Level 8–12 Nurses, Private hospital health workers). Table 1 provides the demographics for the in-depth and focus group participants.

**Table 1:** Demographics of participants for the in-depth interviews and the focus group discussions

Category	Respondents	Gender	Career Level	Other Information
Key Informant Interviews (KIIs)	Decision makers, Programme managers	M/F	Senior positions	Respondents hold managerial positions (strategic level)
In-depth Interviews (IDIs)	Early-, mid-, and senior-career women	F	Early, Mid, Senior	Involved women at different career stages in public health
Focus Group Discussions (FGDs)	4 groups (12 participants each)	F	CHEWs, Nurses, Private hospital workers	4 groups: CHEWs, Nurses (< level 8), Nurses (Level 8–12), Private hospital health workers

*M* Males, *F* Females

The themes and sub-themes are Barriers to Workforce Capacity Development***, ***Enablers of Women’s Participation in Workforce Capacity Development, Strategies to Reduce Workplace Gender-related Barriers (Facilitators) and Recommendations to Reduce Workplace Gender-related Barriers.

### Theme 1: barriers to women’s involvement in public health WCD

Barriers emerged at all SEM levels: individual (e.g., confidence), interpersonal/household (spousal consent, childcare), organisational (leave policies, scheduling, harassment), community (norms, insecurity), and policy/system (implementation gaps despite gender-mainstreaming instruments). Women face inequalities in access to school, employment, and career progression, and gender discrimination in the workplace is more common in low- and middle-income nations [16]. Women continue to face discrimination in workforce capacity development across the world, which limits their access to leadership, mentorship, and training opportunities [17].

#### Socio-cultural norms

According to the desk review, women's educational, employment, and career prospects in Nigeria's healthcare industry are restricted by sociocultural norms, such as early marriage and conventional gender roles [18]. Women are unable to reach their full economic potential due to cultural and societal standards and their expressions. In many civilisations, the desire for boys disadvantages girls in terms of family investment in human capital as well as asset ownership and control [19] [20]. Twenty-two [22] participants talked about socio-cultural norms as a barrier in the qualitative interview

*"There are cultural barriers…"* IDI, Mid-level, Female.

*"...the man will decide your fate as a woman that is why then our cultural belief… they strongly belief that men have upper arm*” KII, Decision Maker, Female

*"There are women who would have opportunities for career advancement but would have to take consent from the husband before they take it, I've seen it before where a woman has an opportunity to go and build capacity …. but the husband is insisting you must do it somewhere else... you know cultural influences*” KII, Decision maker, Female.

*“They are more inclined to their culture, so they show favouritism to people who greet in a certain way. So, me coming from the south, I am giving a scenario, I don’t bend down to greet I just greet so it’s seen as disrespect, so they tend to show favouritism to those who they feel are showing respect by bending to greet* " IDI, Mid-level, Female 

#### Gender stereotyping

From the desk reviews, gender biases were noted, particularly those held by male supervisors, hinder women from fully engaging in health-focused WCD. These biases are rooted in traditional gender roles, where women are seen as caregivers and men as capable of independent travel and work in difficult conditions. These preconceived notions about gender ultimately lead to discrimination, reinforced by widespread gender stereotypes. Fourteen [14] participants talked about gender stereotyping in the qualitative interview

*“So far, the perceived role is that the woman should be under the man... So, I have also experienced it”* Senior-Level Manager, Female

##### Societal expectations and stereotypes 

Often lead to the concentration of women in lower-paying healthcare roles and hinder their advancement in public health careers. Women in low- and middle-income countries face unique challenges, including limited access to education and cultural barrie [4]. This was supported by thirty-four [34] qualitative interviews:

*“In my monitoring and evaluation, I know I faced a lot of stereotypes because the M&E space is male dominated. When we are going to the field, they will say as a woman I don’t think you should travel to this place”* State Mid-Level Manager, Female

#### Gender segregation (n=19)

Nineteen [19] participants talked about gender segregation in the qualitative interview. Apart from possible problems with the rule of law, the difficulties women face in entering and progressing in their careers, getting an education, developing skills, and building professional connections are largely due to societal rules that separate gender [19]. Occupational segregation and discrimination against female health workers were reported [34].

The findings revealed that sick leave, the ability to work, and women's overall health are connected and impact the workplace. Yet, we lack a complete picture of how female biology influences work. This suggests we need more studies on health and work, specifically looking at how it might differ between genders [21].

*“If it is something that she has to go to an area that is inaccessible, we try to look at those contexts and try to see if we could get somebody to go and do it on her behalf” *Decision Maker, Male

*“But if I need to call the women, I will first of all ask them whether they will be able to go”* Decision Maker, Male.

#### Sexual harassment (n=3)

From the desk review, sexual harassment was found as one of the barriers to women. A US study indicates that between 20–50% of female students, and over half of female instructors and staff, face sexual harassment in the classroom. “When working women experience sexual harassment from higher-level men, the consequences are particularly severe. Their job satisfaction plummets, their intention to quit rises, and their organizational commitment diminishes. Furthermore, their health suffers considerably, manifesting as increased depression, emotional exhaustion, and various physical well-being issues—a more pronounced negative impact than harassment by men of equal or lower status [22].

Three [3] participants talked about sexual harassment in the qualitative interview.

*“So, for you to go for that, maybe your boss or... somebody might even be saying I want sex for exchange of this, if you need to go for this one you need to give me sex”* State Mid-Level Manager, Female.

#### Family and domestic responsibilities

Another factor identified as impeding women's involvement in health WCD was marriage and family responsibilities. In the case of female volunteer CHWs, research indicates that they may find it challenging to defend their time spent serving the community because they are required to take care of children and other family members in addition to doing domestic responsibilities [1]. These multiple expectations often lead to conflicts between work and family life, which in turn affect women’s ability to sustain or advance in their careers [26].

"*Women play dual roles not just in the place of work because they are also mothers to children and also wife to a man whom they are going to cook for everyday or do one or two things before coming to work"* KII, Decision maker, Male

*“Actually, most of the challenges I have is combining work with family activities”*State mid-level Manager, Female.

Such family pressures may also force some women to leave their jobs or relocate to accommodate their husbands’ career moves, resulting in the loss of professional experience and career progression opportunities [23]. Moreover, a woman’s decision to enter or remain in the workforce is often shaped by family dynamics, household size, and income level [27].

#### Missed career and trained opportunities

Many participants reported that gendered family obligations frequently prevented women from taking advantage of professional development and training opportunities. These constraints diminish women's value, recognition, and visibility at work, while also restricting access to career-enhancing resources such as mentorship, sponsorship, and capacity-building sessions [24].

*“I have seen a colleague that maybe one or two things in the house made the person not to attend a particular training”*State Mid-Level Manager, Female

*“I have seen instances in government stakeholders where women were unable to attend capacity-building sessions due to family responsibilities” State Mid-Level Manager, Female*

“…. *opportunities will arise, for instance, there are jobs that may be in remote areas or far areas and you as a woman cannot go where men can easily go because you will have to consider other factors because if you move out of where your family is, who is going to take care of your other responsibilities. So yes, there are opportunities that come that you let go, just because of your gender, and other responsibilities* …**.** " KII, Decision maker, Female

Overall, forty-two [42] participants discussed how family responsibilities, gender norms, and domestic expectations collectively limit women’s full participation in the workforce and leadership roles.

#### Competing social and religious expectations

Participants also discussed social and religious expectations that make balancing professional and family life even more difficult.

“*… you have situations where a woman wants go for the PhD and people are telling her that you have a baby you are nursing, you have children in primary school, you have to take care of your husband, you have your role in church, you know there are just different roles and responsibilities demanding your attention, why do you want to add something else*" IDI, Mid-Career, Female

##### Reproductive roles/childbearing

Moreso, because male managers believed that maternal duties reduced women's productivity, women in the health sector were penalized for having children [25].

*“Because, you know, some organisations were like, soon this woman will take in, this woman will have a baby, and I don't know, once you have a baby, you might not really put in your best as they expect you to” *State Mid-Level Manager, Female

*“I recall that we used to have a supervisor, one of the directors or managers that used to say, my work is very delicate. If I bring them now, small time, they’ll tell me they are going on maternity leave. We also even have a female manager who also said, I do not want, no, no, no, no, no, I don't want any person that will come here and be telling us they want to give them maternity leave” *State Mid-Level Manager, Female.

*“Some of them are family. If whatever training you are going for is outside your state, you will find it difficult to go because you don’t want to leave your children behind” *State Mid-Level Manager, Female

*“It will definitely affect recruitment and retention... imagine the lifespan of a five-year project and you have to take maternity leave twice***.***”*Decision Maker, Male

*“Some of the challenges were that having little children and it was a bit difficult sometimes because our work entails a lot of travelling” *State Mid-Level Manager, Female

#### Rural vs urban employment patterns

Gaining diverse experience in rural areas was seen as beneficial, especially by men. They often had fewer family ties that would prevent them from relocating and saw rural postings as a path to future training, invitations to international workshops, and promotions. As a result, human resource managers tended to assign men to very remote locations, expecting them to stay longer and not request transfers [28].

Women's participation in the workforce differs significantly between urban and rural areas. A higher percentage of women in urban areas (55.77%) are employed compared to those in rural areas (40.29%). This disparity is primarily due to the greater availability of job opportunities in cities.

#### Leadership and gender imbalance

Even though women make up most of the public health workforce, men predominantly hold managerial positions. Women are notably underrepresented in leadership and decision-making roles, particularly in county hospitals. A key societal goal is to address this imbalance by increasing the number of women in these influential positions [16].

#### Gender-based discrimination and workplace barriers

Twenty-one [21] participants talked about the Patterns of employment, Discrimination and Male domination of management positions in the qualitative interview.

*“We all are someone’s child not necessarily a woman, you consider timing, in most of the times this our work are not planned it just comes like that without any planning and if you are not careful you can't meet up”* Low-Level Career, Female.

#### Safety and security

Safety concerns and the perception of public areas as"male spaces" significantly restrict women's economic and physical mobility, limiting their freedom of movement [26]. From this study, the increase of insurgency and insecurity was seen to generate fear of mobility and relocation which was a great barrier to women in the public health space. They felt several places were unsafe for them, especially because of their gender.

This is a challenge for women’s participation in capacity development opportunities in public health. Five participants talked about safety and security in the qualitative interview.

“*The network access and the resources, you know, some persons are used to a specific location and who don't want to move. There is also a limitation*”KII, Decision Maker, Male

” *you must give your consent to go wherever the organisation is sending you. So, in a way, because of the physiological should I say makeup of a woman, sometimes the responsibilities in the family, it can be challenging for women to accept such opportunities as easily as a man would* …."IDI, Low-Level Manager, Female

“*The network access and the resources, you know, some persons are used to a specific location and who don't want to move. This is also a limitation*” KII, Decision Maker, Male

Some additional findings were obtained from the desk reviews (that were not gotten from the qualitative interviews done).KII, Decision Maker, Male

There is a Lack of equal opportunities and differences in wages, female employees reported being unhappy with their pay, as they felt they were compensated less than they deserved [19]. This disparity is attributed to the gender pay gap, where women are not given the same opportunities or wages as men [19]. This gap often stems from employers' discriminatory practices, which lead them to believe that female employees are less productive than their male counterparts, even when they have the same education levels. As a result, women end up with lower salaries [26]. Across all situations studied, women were found to be primarily in lower-paying jobs. There could also be differences in other sources of income [23].

Gender inequality and a lack of workplace support systems are significant barriers that prevent women from entering and thriving in the workforce. Many workplaces do not offer the necessary environments for women to effectively manage their responsibilities both at home and on the job. Furthermore, societal expectations that women primarily handle domestic tasks, combined with a scarcity of desirable jobs, negatively affect female employment [26]. Even when women do secure employment, they often face poor working conditions [22]. The lack of recognition and visibility for women's health concerns in the workplace can also hinder their participation [21].

Some additional findings were obtained from the qualitative interviews (that were not seen from the desk reviews done)

#### Inconvenient timing and funding

Short-notice invitations and self-funding requirements were noted as a barrier to women's participation in health WCD.

*“I have had series of opportunities some I have been able to take up and some I have not been able to take up... the limitation is time and funding”* State Mid-Level Manager, Female.

*“I have had instances where women had to pay for their own travel, their child, and a nanny, which can be a financial burden” *Decision Maker, Male

#### Low educational background

Six [6] participants talked about low educational background as a barrier to females in Workforce Capacity Development in the qualitative interview.

*“Because the lead consortium partner was an international organisation, they requested for her credentials. By the time they came back with the credentials, they were just seeing secondary school”* State Senior-Level Manager, Male.

*“The issue is not giving equal opportunity. It's that the number of educated women is far less than the men.”* State Mid-Level Manager, Male.

#### Lack of confidence

Two [2] participants talked about a lack of confidence in themselves in the qualitative interview.

*“They have a lot of them with wonderful credentials. Okay, defend what you have over here. And they couldn't because they lacked that confidence”* Decision Maker, Male

*“Like now I know something but if you ask me, I won't be able to say it because I'm feeling shy, let me not say it”*State Low-Level Manager, Female

### Theme 2: enablers of women’s participation in WCD

Enablers clustered across SEM levels, from mentoring and refresher training (individual/organisational) to supportive spouses and family-friendly practices (household/organisational), community safety, and enabling policies and programmes (policy/system).

#### Gender-related policies and legal instruments

From the desk reviews of Gender-related Policies and practices within the workspace, to assist organisations and government agencies in enhancing gender equality in the public health workplace, the Nigerian government has put in place gender-related policies. The issue is the inadequate application and use of existing laws, such as the Violence Against Women Act [6, 11]. Globally, gender equality is acknowledged as a human right, and several international organisations have pushed for laws to eradicate gender differences in workforce development [29].

International Conventions on Elimination of all Forms of Discrimination Against Women (ICEDAW) emphasises the importance of gender-related policies in achieving gender equality [28, 30].

Nigeria has implemented several policies to advance women's rights. The Universal Basic Education (UBE) Act, based on the 1999 Constitution, provides free primary and junior secondary education for all, without gender bias, significantly boosting girls' school attendance [31]. The Trafficking and Enforcement Act of 2015 aims to protect human dignity, prevent violence and exploitation, and combat human trafficking, particularly involving women and children, by establishing both preventative measures and support systems for victims [30]. Lagos State's Domestic Violence Law No. 15, 2007, offers broad legal protection against various forms of abuse for women and girls [31].

The INEC Gender Policy, revised in 2021, has raised awareness about gender issues in elections and the need to overcome systemic and patriarchal obstacles to political participation [26]. Nigeria's National Gender Policy, which replaced the previous policy on women, seeks to empower women and achieve gender equality, aligning with several international and regional protocols [32].

Furthermore, public service rules and the Labour Act of 2014 provide maternity leave benefits for pregnant and nursing female employees, including paid leave and protection against job termination due to childbirth-related issues [31, 33]. The Labour Act also grants nursing mothers breaks to care for their children [27, 33].

However, certain aspects of Nigerian law disadvantage women. The constitution's use of masculine language and the lack of provisions addressing this in Section 318 imply an unequal status for women [34]. Section 26:2, for instance, allows Nigerian men to transfer citizenship to their spouses but denies this right to Nigerian women. Additionally, the Police Act (Sections 127 and Regulation 124) prevents married women from joining the police force, mandates the discharge of pregnant unmarried policewomen, and requires female officers to obtain approval before marrying [35].

#### Zero-tolerance and anti-harassment policies (interview evidence)

Qualitative findings from five [5] stakeholder’s consultations noted that there is zero tolerance for Sexual Harassment

*“The ‘do no harm’ policy has really helped us in the workplace whereby, no matter who you are, no matter your position in [XYZ organisation], if you try to bring that sexual harassment against any of your female staff, those cases are being picked up and sanctions will be given to you”* Senior-Level Manager, Female

*“Sexual harassment happens and is something that [XYZ organisation] are taking it as a strong hold and they implement these policies against any form of harassment or sexual exploitation of anyone” *Senior-Level Manager, Female

#### Family-friendly workplace supports

From the qualitative interviews, there were also workplace considerations and support for women

* “We make things family-friendly, allowing women to bring their children to training and covering child-related expenses” *State Senior-Level Manager, Male

*“Organisation XYZ has been a little bit flexible for the female colleagues to work at their own pace” *Decision Maker, Male

*“We have an anti-harassment policy that protects women from exposures that might hinder their participation” *Decision Maker, Male.

#### Gender balance & mainstreaming within HRH

There are also policies on Gender Balance

* “But WHO has already marked a period in which a woman is expected to go on leave. Because in as much as she wants to work, she needs to have a healthy delivery too. And… For the WHO, family come first and that has been entrenched in us” *State Senior-Level Manager, Male

*“We have policies on human resources for health, and that policy does not discriminate. It is a holistic policy that provides guidelines on capacity building for all workers, either male or female”* State Mid-Level Manager, Female

*“Yes, I know we have a gender policy that is domiciled in the division of GASH. This policy promotes equitable access of everyone either male or female to basic services including capacity building” *State Mid-Level Manager, Female

*“I will say we have a lot of policies that support this work-life balance... we have a parental leave policy for men, that is the 14 working days, and then we have the maternity leave for women”* State Senior-Level, Female

*“We have now developed a gender strategy... to ensure that there is gender mainstreaming across all work streams, including capacity-building* programmes” Decision Maker, Male

### Theme 3: strategies to reduce workplace gender-related barriers (facilitators)

Proposed strategies targeted multiple SEM levels simultaneously; awareness and mentoring (individual), childcare supports and flexible work (household/organisational), safer field practices (community), and operationalisation of national programmes and HR rules (policy/system).

#### Awareness and sensitization

The desk review identified ideas for lowering gender-related obstacles in the workplace. Programs for raising awareness of gender policies and household duties are available. Strategies to lessen gender-related hurdles were identified, including the implementation of gender policies in the workplace, awareness campaigns, workplace mentorship programs, and scholarships for women. These deal with cultural norms that are crucial to removing these obstacles to women's involvement in WCD[36, 37].

Some programmes have proved efficacy in wedging these gender-related barriers.

#### Gender mainstreaming and government programmes

Gender mainstreaming is a government method for improving decision-making so that policies and budgets promote gender equality. In Nigeria, several national programmes embed gender concerns to expand women’s access to resources, skills, services, and decision-making spaces such as:

##### Agricultural and economic policy levers (ERGP/green alternative)

Nigeria’s Green Alternative, an agricultural policy within the Economic Recovery and Growth Plan (2017–2020), builds on the Agricultural Transformation Agenda[38]. It aims to work with stakeholders to grow a resilient agribusiness sector that secures food, increases exports, and creates jobs and incomes. Respondents linked this policy to practical gains for women, especially through improved access to land, facilities, and finance, which supports inclusive development. They also pointed to micro-loans from the Government Enterprise and Empowerment Programme (GEEP) and the Women Empowerment Fund. Under the ERGP’s education strategy, girls’ schooling and infrastructure are priority areas, while the Conditional Cash Transfer Programme continues to target the poorest households, mainly through women in the Social Register.

##### Digital inclusion for girls and women (girls in ICT)

The Girls in ICT Training [39] helps women and girls build digital skills and contribute to the economy. Launched by the Ministry of Communication Technology with HUAWEI as a training partner and supported by the Youth Alliance on ICT for Development, the programme has trained over a thousand participants in Lagos and Abuja. Participants described it as a pioneering effort that is closing the ICT gender gap in West Africa, with some trainees gaining international scholarships or employment at HUAWEI.

##### Microfinance for women’s enterprise (market moni/GEEP)

 Market Moni, part of GEEP and managed by the Bank of Industry, provides micro-financing. While more than 24,000 individuals are reported to have benefited, respondents stressed the need for a focused review to verify how many women have been reached since inception.

##### Connectivity and rural access (USPF)

The Universal Service Provision Fund (USPF) advances the government’s goal of making ICT available in rural, underserved, and remote communities [40]. Participants viewed last-mile connectivity as a practical complement to mainstreaming because it enables women to access training, deliver services, and participate in digital work.

##### Support for continuous professional development (CPD): organisational practices

Findings show that day-to-day organisational practices can make gender mainstreaming tangible.

- Peer mentoring and coaching. Participants described structured, peer-to-peer mentoring that links colleagues within the same unit and career path to someone more experienced. As one female participant in a state-level focus group put it, “People within the same unit and people within the same career path are linked to each other, usually someone who has more experience.”
- Regular refresher training. Managers reported routine refreshers to maintain skills, even during tight funding cycles. A state mid-level female manager explained, “We call them on a quarterly basis, and we do a refresher training whether there is fund or no fund.”
- Family-friendly leave and HR support. Respondents described formal leave and practical help that make participation feasible after childbirth. A senior-level female manager noted, “After delivery… we give them enough period for… maternity leave to take care of their children.” Another senior-level male manager added, “There are funds allocated for making sure that if you have a house help that can help you.”
- Field supervision and skill application. Teams use home visits to reinforce learning and ensure that capacity built in training is applied in communities. A senior-level female manager explained that home visits help verify that “the capacities… built into our staff are implemented in the community.”

### Theme 4: recommendations to reduce workplace gender-related barriers

Recommendations therefore emphasised multi-level bundles: capacity building plus childcare and flexible scheduling, embedded in clear organisational rules and backed by enforceable policy and dedicated budgets. The desk review indicates that an enabling environment is essential for gender equality and for advancing women in the health sector, and that progress requires a combination of mutually reinforcing strategies [41].

#### Create enabling environments via policy making

Participants emphasised the role of HR and leadership in removing institutional obstacles. They argued that professional women, working with HR and allies in leadership, can shape policies that advance equity and embed fair processes for selection, deployment, and development.

#### Engage men and families

Findings point to the importance of engaging men and spouses so that caregiving and maternity are anticipated rather than penalised. As one senior-level female manager said, “Men in the workplace also need sensitisation… she’ll get pregnant, she’ll give birth, and she’ll come back to work.” Another state mid-level female manager recommended spousal orientation at onboarding, so families understand work expectations in advance. Where civil service rules require travel with a spouse, respondents asked that provision be made to enable this travel.

#### Invest in workplace infrastructure and flexibility

Respondents highlighted specific investments that directly affect participation.

- *On-site childcare and nursing spaces.* A decision maker explained that a nursery or creche “will go a long way in encouraging women.” A state mid-level female manager recommended a policy requiring MDAs and private organisations employing women to provide a creche and a nanny.
- *Childcare financing and practical supports.* Managers proposed dedicated resources for child minders so women can focus at work without worry: “Allocating resources for support, like child minders, can greatly facilitate women’s participation,” said a state mid-level manager. Others suggested setting aside funds for women with additional family burdens and ensuring practical help so a woman “has a nanny without any worries because they have provided what they need.” Several noted existing allocations to support house help.
- *Health and lactation-friendly facilities.* A state mid-level female manager recommended establishing a small clinic within ministry premises to support parents and reduce out-of-pocket costs.
- *Training logistics and travel flexibility.*Participants suggested reserving accommodation so mothers can attend training with a nanny and, where appropriate, assigning a caregiver to travel with eligible staff.

#### Universal paid family leave (with subsidised childcare)

Equal leave for new parents at all staff levels was seen as a levelling measure so that no group is viewed as a higher cost. Subsidised childcare was viewed as a practical incentive for continued participation. As one senior-level female manager stated, “The whole of society must find a way to accommodate and compensate for the gaps that occur when women take time off for childbirth.”

#### Strengthen policymaking and implementation

Respondents urged moving beyond policy statements to implementation that anticipates hidden costs such as missed commissions or incentives. They also called for enforceable rules. A senior-level female manager remarked that a clear law“would go a long way” because “whoever comes is aware this is the law.” Another recommended transparency in nominations, with a policy to ensure that at least 30–40 percent of nominees are women.

#### Flexible and remote work, and breastfeeding support

Participants endorsed temporary remote or adjusted work during maternity, with clear deliverables, and explicit time for exclusive breastfeeding. A state mid-level female manager suggested virtual work platforms during leave, while a low-career female participant called for a policy that gives time to breastfeed.

#### Policy awareness and women’s education

Finally, participants stressed policy literacy and long-term pipeline investments. Senior managers recommended educating women about existing policies so they can assert their rights and opportunities and encouraged broader awareness. Others highlighted the importance of girls’ education to increase women’s participation over time.

#### Summary of interview-based recommendations

Across interviews, suggested actions included workplace mentorship, stronger policymaking and implementation, policy sensitisation, financial supports for women, flexible and supportive work environments, gender sensitisation for men, socio-cultural policy measures, nursing-mother-friendly policy, parental leave, creche access, and practical travel supports.

## Discussion

Framing the findings within the Social–Ecological Model clarifies that single-point interventions are insufficient to address the barriers women face in workforce capacity development (WCD). Individual skills and confidence are constrained by household responsibilities and permission structures, which are further shaped by organizational practices, community norms, and the broader policy environment. Effective action therefore requires bundled, multi-level interventions—combining, for example, training and mentoring at the individual level, childcare and flexible scheduling at the household and organizational level, safe deployment policies and community engagement at the community level, and enforceable gender-mainstreaming provisions with financing at the policy and systems level. Our study contributes to the growing evidence that women in Nigeria and other low- and middle-income countries (LMICs) continue to encounter significant gender-related barriers in health workforce capacity development. From both desk reviews and stakeholder interviews, we found that socio-cultural norms, early marriage, and conventional gender roles restrict women’s access to education, training, and professional growth. These findings align with previous studies in Nigeria and Egypt that highlight sociocultural restrictions as the most prominent barriers for female health workers [42, 43]. This contrasts with findings from the US and UK, where challenges were often linked to workplace stress and inadequate maternity support. For instance, hospitals were poorly equipped for expectant doctors, and program directors sometimes became antagonistic toward pregnant trainees [44, 45].

The issue of limited female representation in leadership and decision-making positions in Nigeria was a recurring theme in our findings. This resonates with the work of Ayaz et al. (2021), who noted barriers related to women’s underrepresentation in leadership roles [25], and Dhatt et al. (2020), who observed that women in LMICs face unique challenges from cultural barriers and limited educational access [5]. Even in contexts where women dominate numerically in professions such as nursing and midwifery, their presence in management and policy roles remains limited [40]. Globally, this pattern is consistent across sectors, with women underrepresented in senior leadership positions, including manufacturing (24.6%), agriculture (23.3%), and infrastructure (16.1%) [24].

Marriage, family obligations, pregnancy, and childbearing emerged in our study as critical constraints on women’s professional development. Similar observations have been made in Korea and Japan, where cultural expectations around childbearing and caregiving roles affect women’s career planning, transitions, and self-confidence [46, 47]. In our context, safety concerns and insecurity further compounded these challenges, with women perceiving many postings as unsafe due to insurgency or gender-based risks [26]. Such safety considerations often limit women’s willingness or ability to relocate, restricting their career mobility.

Applying an intersectional lens, our findings also highlight disparities between urban and rural women. Rural-urban gaps in labour force participation are evident, with urban women participating at a rate of 55.77% compared to 40.29% for rural women [24]. Urban women benefit from greater opportunities, while rural women face more rigid cultural barriers. Enhancing rural education has been suggested as a means to increase employability and narrow these gaps [24, 27].

Our results further revealed a mismatch between available policies and their implementation. While Nigeria has gender-related policies—including the National Gender Policy Act, the Violence Against Persons Act, and the Economic Recovery and Growth Plan [48]—practical enforcement remains weak. Stakeholders acknowledged the existence of these frameworks but reported persistent discrimination, harassment, and limited institutional support for women. Similar implementation challenges have been documented globally and across LMICs [13, 49]. However, South Africa demonstrates that stronger legal and institutional frameworks can more effectively advance gender equality [47].

The practical implications of these findings are clear. To minimize barriers, Nigeria must move beyond policy formulation toward robust implementation. Awareness campaigns and sensitization about existing gender policies, financial incentives for women, and supportive workplace structures such as childcare facilities, breastfeeding rooms, and flexible scheduling were repeatedly highlighted by stakeholders as critical enablers. Mentorship and supportive supervision should be institutionalized to strengthen women’s confidence and career progression [47]. Moreover, investments in universal paid family leave, subsidized childcare, and equitable deployment policies could significantly reduce structural disadvantages. Importantly, interventions should engage men as allies in women’s empowerment, as studies in Nigeria and elsewhere have shown that male involvement can help transform entrenched negative attitudes [50].

Despite the strengths of our study—particularly its engagement with diverse stakeholders across Nigeria’s geopolitical zones and its capture of women’s first-hand experiences—certain limitations should be acknowledged. First, some potential participants declined to engage due to fear of repercussions or lack of trust. Second, gender barriers vary considerably across cultural, social, and economic contexts, making it difficult to generalize findings across Nigeria or LMICs broadly. Finally, as with most qualitative research, the potential for subjectivity and socially desirable responses cannot be excluded.

Future research should explore the effectiveness of specific gender-sensitive interventions—such as subsidized childcare, mentorship programs, or community engagement initiatives—in improving women’s participation in WCD. Longitudinal studies that track the career trajectories of women in the health workforce would also be valuable in understanding how gender-related barriers evolve over time. Comparative studies between Nigerian states or between Nigeria and countries with stronger gender equality frameworks could generate further lessons for policy and practice.

## Conclusion

Women's participation in health WCD is hindered by gender stereotyping, socio-cultural and political barriers and poor implementation and practice of established policies. Thus, there is a need to address these challenges by incorporating strategies such as sensitisation and awareness campaigns to promote and implement these available policies and limit socio-cultural norms. In addition to these, financial incentives, a supportive environment and workplace mentorship to participate in WCD to reduce workplace gender-related barriers ought to be included.

Furthermore, equal opportunities and maximum engagement of women should be ensured by advocating for women to occupy focal positions. In addition, opportunities should be created to assess social and welfare support such as support for travel with spouse and children or a childminder, creating a conducive work environment, motivation, capacity development and advancement. This would go a long way towards eliminating these barriers.

## Supplementary Information

Supplementary Material 1

## Data Availability

We confirm that the data stored in the repository is easily accessible to others and can be accessed.

## Abbreviations

APPRRW: African Protocols on People’s Rights and the Rights of Women
BPFA: Beijing Platform for Action
CHEWs: Community Health Extension Workers
CEDAW: Convention on the Elimination of All Forms of Discrimination Against Women
FGDs: Focus Group Discussion
GEEP: Government Enterprise and Empowerment Programme
IDI: In-depth Interviews
ICPD PoA: International Conference on Population Development Plan of Action
KII: Key informant Interviews
LMIC: Low and middle-income countries
NEPAD: New Partnership for African Development
OECD: Organisation for Economic Cooperation and Development
PHS: Public Health System
SDG: Sustainable Development Goals
UHC: Universal Health Coverage
UN: United Nations
USPF: Universal Service Provision Fund
WCD: Workforce Capacity Development
WHO: World Health Organisation
WTO: World Trade Organisation
