# Supplementary material for: Exploring gender factors affecting women’s involvement in public health capacity development programmes in Nigeria
Source: BMC Public Health. 2025 Nov 21;25:4097. doi: 10.1186/s12889-025-25438-6 (PMC12639985; doi:10.1186/s12889-025-25438-6)
Supplement: Supplementary file 1 — Supplementary Material 1 [file 12889_2025_25438_MOESM1_ESM.docx]

# **Interview Guide – Exploring Gender Factors Affecting Women's Involvement in Public Health Capacity Development Programmes in Nigeria**

This semi-structured interview guide was developed specifically for this study to explore gender-related factors influencing women's participation in public health capacity development programmes in Nigeria.

# **Section A: Key Informant Interview Guide for Decision-makers**

**Introduction:**

My name is ____ and I am meeting with you on behalf of Corona Management Systems. We are assessing gender-related factors influencing participation in workforce capacity development for women in public health practice in Nigeria. The assessment aims identify any gender-related limitations that hamper participation in workforce capacity development programmes for women in public health.

In view of this, we would like to interview you, to help us gain better understanding on this issue. This will last for approximately one hour. You do not have to answer all the questions and may stop at any time without penalty. Your responses are entirely confidential and will only be shared with the team involved in this assessment. We will keep your responses anonymous and any personal information collected during this interview such as your name and phone number will be kept in a password-protected document in a password-protected device. We wish to record this interview strictly for transcription purposes after which the recordings will be destroyed. However, if you do not wish to be recorded, notes will be taken instead.

Do you agree for this interview to go forward? Do I have your permission to record this interview?

| 1. In your organisation, do you feel that women have equal opportunity to be recruited or retained?   **Probes:** Do you feel that there any issues that contribute to women not being recruited or retained in your organisation, or factors that sometimes slow down career progression for them? |
| --- |
| 1. What are some of the social and cultural barriers in Nigeria that you feel potentially limit women’s participation in workforce development programmes?   **Probes:** How about women from minority ethnic groups, do they face any additional barriers participation in workforce development programmes? Do you feel that these limitations currently affect career progression? |
| 1. How does your organisation ensure that workplace capacity building activities benefit the women that work in the organisation?   **Probes:** Are there any additional provisions to ensure that women participate? Is there a policy? If not, has enacting a policy been considered? If you have a policy, what are some of the key provisions? |
| 1. How does your organisation track capacity building interventions that your workers participate in?   **Probes:** How do you keep track of people’s learnings and development in line with the competencies that they are expected to have? Do you have competency framework for each position in your organisation? Do people undertake trainings in line with a competency framework? Does tracking of capacity building disaggregate information on participants by gender? |
| 1. When your organisation implements capacity building interventions, how do you make sure that women in the organisation are using and benefiting from these interventions as much as men?   **Probes:** Do you analyse participation in these capacity building activities? If yes, do you specifically look at how well the women in the organisation are participating? Are the women getting similar numbers of opportunities? Are the women taking the opportunities as much as the men? Are these opportunities designed in any way as to support women to participate as much as men? |
| 1. Are there any practices that potentially limit women’s participation in your organisation?   **Probe:** Do you feel that there are any issues that contribute to women in your organisation not participating in capacity building opportunities? What about for other organisations working in public health, do you know of any issues that contribute to same? What are some of approaches that you have taken to encourage women in your organisation to participate in capacity building opportunities as much as the men? Do women get mentorship? Any additional provisions made for women of child-bearing age? Why not? |
| 1. What are some of the other approaches that you think can work to encourage women to participate in capacity building opportunities as much as the men?   **Probe:** What are some of approaches that you have taken to encourage women in your organisation to participate in capacity building opportunities as much as the men? Do women get mentorship? Any additional provisions made for women of child-bearing age? Why not? |
| 1. What are your thoughts on allocating targeted resources to ensure that women are able to participate in capacity building opportunities as much as the men?   **Probes:** What do you think that allocating these kinds of targeted resources can accomplish? Do you think that resources allocated to support travel and meals for a spouse or a child minder for women with young children can be helpful? |

# **Section B: Key Informant Interview Guide for Managers and Capacity-building Providers**

**Introduction:**

My name is ____ and I am meeting with you on behalf of Corona Management Systems. We are assessing gender-related factors influencing participation in workforce capacity development for women in public health practice in Nigeria. The assessment aims identify any gender-related limitations that hamper participation in workforce capacity development programmes for women in public health.

In view of this, we would like to interview you, to help us gain better understanding on this issue. This will last for approximately one hour. You do not have to answer all the questions and may stop at any time without penalty. Your responses are entirely confidential and will only be shared with the team involved in this assessment. We will keep your responses anonymous and any personal information collected during this interview such as your name and phone number will be kept in a password-protected document in a password-protected device. We wish to record this interview strictly for transcription purposes after which the recordings will be destroyed. However, if you do not wish to be recorded, notes will be taken instead.

Do you agree for this interview to go forward? Do I have your permission to record this interview?

| 1. What policies and guidelines exist in your organisation that speakparticipation of women in workforce capacity development programmes in public health practice?   **Probes:** If you have a policy or guideline, what are some of the key provisions or issues that it addresses? Are the policies being implemented? Do you feel there is a need for policies and guidelines to exist? In your day- to- day activities, what efforts are made to ensure women participate better? Are there informal steps that are taken to support women to participate in these programmes even though there is no specific policy or guideline? |
| --- |
| 1. How do you track capacity building interventions that you provide or that your team members participate in?   **Probes:** Do people undertake, or do you provide trainings in line with a competency framework? How do you keep track of people’s learnings and development in line with a competency framework? Does tracking of capacity building disaggregate information on participants by gender? In your day-to-day activities, what measures put in place to encourage women are actually implemented/monitored? |
| 1. Are the women getting similar numbers of opportunities as men? Are they actually taking these opportunities as much as the men? Are these opportunities designed in any way as to support women to participate as much as men?   **Probe:** When your team members participate in or you provide capacity building interventions, how do you make sure that women in the organisation are using and benefiting from these interventions as much as men? |
| 4Does this organization take preference in recruiting men to women? What do you think are the issues that contribute to women not being recruited or retained, or that sometimes slows down career progression for them?  **Probe**: Are women of childbearing age considered in employment? Is it a deterrent due to maternal leave? Are measures put in place to support women and the disabled like provision of a creche for child bearing women? Any spare room for breast feeding mothers? How about women from minority ethnic groups, do they face additional barriers to career advancement in public health? |
| 5. Do you feel that there any issues that contribute to women not participating in capacity building opportunities?  **Probes:** Are there any practices that can potentially limit women’s participation in your organisation? What are some of approaches that you have taken to encourage women in your organisation to participate in capacity building opportunities as much as the men? Do women get mentorship? |
| 6. What are your thoughts on allocating targeted resources to ensure that women in public health are able to participate in capacity building opportunities as much as the men?  **Probes:** What do you think that allocating these kinds of targeted resources can accomplish? Do you think that resources allocated to support travel and meals for a spouse or a child minder for women with young children can be helpful? |
| 7. What are some of the approaches that you have taken to encourage women to participate in capacity building opportunities as much as the men?  **Probes:** Any measures put in place for implementation in your organization? |
|  |

# **Section C: In-depth Interview Guide for Senior-career Women in Public Health**

**Introduction:**

My name is ____ and I am meeting with you on behalf of Corona Management Systems. We are assessing gender-related factors influencing participation in workforce capacity development for women in public health practice in Nigeria. The assessment aims identify any gender-related limitations that hamper participation in workforce capacity development programmes for women in public health.

In view of this, we would like to interview you, to help us gain better understanding on this issue. This will last for approximately one hour. You do not have to answer all the questions and may stop at any time without penalty. Your responses are entirely confidential and will only be shared with the team involved in this assessment. We will keep your responses anonymous and any personal information collected during this interview such as your name and phone number will be kept in a password-protected document in a password-protected device. We wish to record this interview strictly for transcription purposes after which the recordings will be destroyed. However, if you do not wish to be recorded, notes will be taken instead.

Do you agree for this interview to go forward? Do I have your permission to record this interview?

| - 1. 1. Are there policies that aid or prevent participation of women in workforce capacity development programmes in public health practice?   **Probes:** Did any of these policies encourage or mar your success as you climbed the ladder in your career? Can you expatiate mor on this? If you do not know about any, do you feel the need to have one? If there was no policy, do you feel that the absence of these policies and guidelines have impacted on your career progression? Are there informal steps that you know are taken by some organisations to support women to participate in these programmes even though there is no specific policy or guideline? |
| --- |
| 2. In the progression of your career what were the challenges you had and the successes? Did you raise a family whilst balancing your career and how did you manage?  **Probe**: Throughout your career, how did the organisations that you have worked with make sure that women in the organisation were using and benefiting from capacity building interventions as much as men? Were the women getting similar numbers of opportunities and not taking them as much as the men? Were these opportunities designed in any way as to support women to participate as much as men? |
| 3. Do you feel that there any issues that contribute to women not being recruited or retained, or that sometimes slows down career progression for them?  **Probe**: Did you have challenges in your recruitment? Did you have to perform far above your male counterparts for you to be chosen? Were there any form of discrimination? Did you directly experience any of these issues? Are you happy to shed more light on these issues, if you experienced them, and share some lessons about how you thrived and what could have been done differently? |
| 4. What do you think we need to put in place for the next generation of women to thrive in their families and in their professional spaces, taking advantage of every capacity building opportunity?  **Probe:** I am curious to know how you managed with capacity building opportunities throughout your career. Did you go to every training that was available? Did you skip some? How often did you skip some because of your family or because you did not have help? How have your thoughts on gender-related barriers to workforce capacity development in public health practice evolved throughout your career? Do you feel like your career could have progressed quicker, if you took every opportunity? Was there any stage when you felt such barriers did not exist? Do you feel like the onus is on the individual woman to overcome these barriers by herself? |
|  |
| 1. What are your thoughts on allocating targeted resources to ensure that women in public health are able to participate in capacity building opportunities as much as the men?   **Probes:** What do you think that allocating these kinds of targeted resources can accomplish? Do you think that resources allocated to support travel and meals for a spouse or a child minder for women with young children can be helpful? |
| 1. What are some of approaches that you have taken to encourage women to participate in capacity building opportunities as much as the men?   **Probes:** How was the support system that encouraged you to make it to the top in your career? Were there provisions/extra space/room to bring in your baby to work if the need arises? |
| 1. What are your thoughts on allocating targeted resources to ensure that women are able to participate in capacity building opportunities as much as the men?   **Probes:** What do you think that allocating these kinds of targeted resources can accomplish? Do you think that resources allocated to support travel and meals for a spouse or a child minder for women with young children can be helpful? |

# **Section 4: In-depth Interview Guide for Early- and Mid-career Women in Public Health**

**Introduction:**

My name is ____ and I am here with my colleague, _______. We are meeting with you on behalf of Corona Management Systems. We are assessing gender-related factors influencing participation in workforce capacity development for women in public health practice in Nigeria. The assessment aims identify any gender-related limitations that hamper participation in workforce capacity development programmes for women in public health.

In view of this, we would like to have a discussion with you, to help us gain better understanding on this issue. This will last for approximately one hour. You do not have to answer all the questions and may stop at any time without penalty. Your responses are entirely confidential and will only be shared with the team involved in this assessment. We will keep your responses anonymous and any personal information collected during this interview such as your name and phone number will be kept in a password-protected document in a password-protected device. We wish to record this discussion strictly for transcription purposes after which the recordings will be destroyed. However, if you do not wish to be recorded, notes will be taken instead.

Do you agree for this discussion to go forward? Do I have your permission to record this discussion?

| - 1. Are there policies in your organization concerning women in workforce?   2. **Probes:** If you know of any policies or guidelines, what are some of the key provisions or issues that it addresses? If there is none, do you feel the need to have one? Have these policies made or marred you in the past? If there are no policies,, do you feel that the absence of these policies and guidelines have impacted on your career progression? How long does your maternity leave last, if you have/had any? Tell us about it |
| --- |
| - 1. So far in your career, how do the organisations that you have worked with or are working with make sure that women in the organisation are using and benefiting from capacity building interventions as much as men?   **Probes:** What are your past limitations to utilising capacity building opportunities? What are your current participation in capacity building opportunities, and what are your future aspirations for capacity building and career progression? Are the women getting similar numbers of opportunities as much as the men? Are these opportunities designed in any way as to support women to participate as much as men? Are women taking those opportunities? |
| 3. Do you feel that there are issues that contribute to women not being recruited or retained, or that sometimes slows down career progression for them?  **Probes**: During your recruitment, were you marginalized? Have you experienced any form of ethnic or tribal discrimination? Have you directly experienced any form of discrimination so far? Are you happy to shed more light on these issues, if you experienced them, and share some lessons about how you thrived and what could have been done differently? |
| - 1. Do you feel that there any issues that contribute to women not participating in capacity building opportunities?   **Probes:** **:** Are there any practices that you feel contribute or limit women’s participation in your organisation Have you directly experienced any of these issues? Are you happy to shed more light on these issues, if you experienced them, and share some lessons about how you thrived and what could have been done differently? |
| 5. What do you think are the reasons women do not participate in career development opportunities?  **Probe:** Do you feel like your career could have progressed quicker, if you took every opportunity? I want to know what you would have liked to help you take every career development opportunity. What do you think we need to put in place for the next generation of women to thrive in their families and in their professional spaces, taking advantage of every capacity building opportunity?  Was there any stage when you felt such barriers did not exist? Do you feel like the onus is on the individual woman to overcome these barriers by herself? |
| - 1. What are your thoughts on allocating targeted resources to ensure that women in public health are able to participate in capacity building opportunities as much as the men?   **Probes:** What do you think that allocating these kinds of targeted resources can accomplish? Do you think that resources allocated to support travel and meals for a spouse or a child minder for women with young children can be helpful? Have you backed down on a career training because of lack of help for your child? Tell us more about that?.. |
| - 1. What are some of the approaches that you have taken to encourage women to participate or taken yourself in capacity building opportunities as much as the men?   **Probes:** Are you having social support and a balanced work-life? Do you have children? Are there measures to encourage or support you like a breastfeeding room? A room for toddlers? Is your maternity leave enough? Even when you come back to work, do you close the same time as your other colleagues? What recommendations will you make to support women especially during their child bearing age? |
|  |
